# Mastopexy Strategies for Ptotic Breasts in Patients Choosing Autologous Reconstruction Following Prophylactic Mastectomy

**DOI:** 10.3390/jcm12093082

**Published:** 2023-04-24

**Authors:** Charalampos Varnava, Miriam Bogusch, Sascha Wellenbrock, Tobias Hirsch, Philipp Wiebringhaus, Maximilian Kueckelhaus

**Affiliations:** 1 Division of Plastic Surgery, Department of Trauma, Hand and Reconstructive Surgery, University Hospital Muenster, 48149 Muenster, Germany; 2 Department of Plastic, Reconstructive and Aesthetic Surgery, Hand Surgery, Fachklinik Hornheide, 48157 Muenster, Germany; 3 Institute of Musculoskeletal Medicine, University Hospital Muenster, 48149 Muenster, Germany

**Keywords:** breast reconstruction, DIEP, microsurgery, mastopexy, ptosis, post bariatric

## Abstract

Background: Autologous breast reconstruction is a reliable solution for many patients after mastectomy. While this technique represents a standardized approach in many patients, patients with ptotic breasts may require a combination of procedures to achieve an aesthetically pleasing result. Methods: We reviewed the mastectomy and free-flap breast reconstruction procedures performed at our institution from 2018 to 2022 in patients with ptotic breasts. The technique used to address the ptosis was put in focus as we present the four strategies used by our reconstructive surgeons. We performed two different one-stage and two different two-stage procedures. The difference between the two-stage procedures was the way the nipple areola complex was treated (inferior dermal pedicle or free skin graft). The difference between the one-stage procedures was the time of execution of the mastopexy/breast reduction (before or after the mastectomy and autologous breast reconstruction). Results: The one-stage procedure was performed with a free NAC in three patients and with a pedicled NAC in five patients. The two-stage procedure was performed in seven patients, with six of them undergoing mastopexy before and one patient undergoing mastopexy after the bilateral mastectomy and autologous reconstruction. No flap loss or total loss of the nipple areola complex occurred. Partial NAC loss was observed in five breasts in the single-stage group without any occurrence in the double-stage group. Conclusions: While both one- and two-stage procedures were performed in a safe fashion with satisfactory results at our institution, larger trials are required to determine which procedure may yield the best possible outcomes. These outcomes should also include oncological safety and patient-reported outcomes.

## 1. Introduction

There is an increasing number of identified BReast CAncer (*BRCA*) *1* or *2* pathogenic variant (PV) carriers [1]. This can be attributed to universal genetic tumor testing in ovarian cancer patients [2], better diagnostic tools, increased referral rates, and patient awareness. The demand for prophylactic mastectomy in such patients leads to an increase in the need for breast reconstruction procedures.

Hartmann et al., showed that prophylactic mastectomy can considerably reduce the incidence of breast cancer in women with a high risk of breast cancer based on family history [3]. The superiority of nipple-sparing mastectomy (NSM) compared to skin-sparing mastectomy (SSM) regarding psychosocial and sexual well-being, body image, feeling of mutilation, and satisfaction with the appearance of the nipple has been demonstrated previously [4,5,6]. It should also be noted that there are studies which do not show any significant difference between NSM and SSM with reconstruction of the nipple areola complex (NAC) [7]. 

The complexity of surgery planning increases in patients with ptotic breasts requiring a mastectomy, as the surgical management of these patients often requires extensive tissue removal and reconstruction. Mastectomy with autologous reconstruction has emerged as a viable surgical approach in such cases, as it allows for the removal of breast tissue while preserving a natural breast shape and contour. In the literature, reconstruction of ptotic breasts is often associated with increased surgical complication rates, particularly for NAC necrosis [8,9]. These complications can result in poor surgical outcomes, such as asymmetry, poor cosmetic results, and patient dissatisfaction. As a result, the management of large and ptotic breasts requires specialized surgical skills and expertise. However, some authors argue in favor of performing reconstructions regardless of breast characteristics using several algorithms [10].

A modified nipple-sparing mastectomy with a periareolar pexy has been described for women with medium-sized breasts [11]. Women with large and ptotic breasts represent a challenging subgroup of patients. A thorough understanding of surgical techniques, outcomes, and associated complications is necessary to achieve optimal surgical outcomes in these patients. The aim of a reconstructive surgeon is not only to restore the lost volume and skin after mastectomy, but also to provide an aesthetically pleasing result. This can be a difficult task, especially in cases where a mastopexy is necessary. Similar to augmentation mastopexy, special attention must be given to preparing an accurate surgical plan. In ptotic breasts, the surgeon has the choice to perform the augmentation and the mastopexy as a one-stage or as a two-stage procedure. The same applies to breast reconstruction procedures in ptotic breasts.

Patients with large and ptotic breasts or post-bariatric patients with ptotic breasts who undergo a breast reconstruction after mastectomy, either due to breast cancer or prophylactically, are candidates for these kinds of procedures. The reports on autologous breast reconstruction of large and ptotic breasts found in the literature are limited and there is no clear recommendation on which procedure to use.

This paper aims to review the current literature on prophylactic mastectomy and autologous reconstruction in patients with large and ptotic breasts. It also aims to describe our experience with four options of autologous breast reconstruction using the DIEP flap after prophylactic mastectomy in this challenging patient population, with a focus on surgical techniques and associated complications. 

## 2. Materials and Methods

This study was approved by the local ethics committee.

We conducted a retrospective study by reviewing the electronic medical records of our institution. We reviewed the mastectomy and breast reconstruction procedures with a free flap performed at our institution between 2018 and 2022. All patients with ptotic breasts grade 2 and 3 after Regnault [12] who underwent a mastectomy, autologous breast reconstruction with DIEP flap and breast reduction or mastopexy were selected for further analysis and were included in the study. The technique used to address the ptosis was put in focus and we present each procedure used by our reconstructive surgeons. We provide details regarding the patient characteristics, surgery, and postoperative complications.

### 2.1. Indications for the Planned Procedures

**Figure 1:**
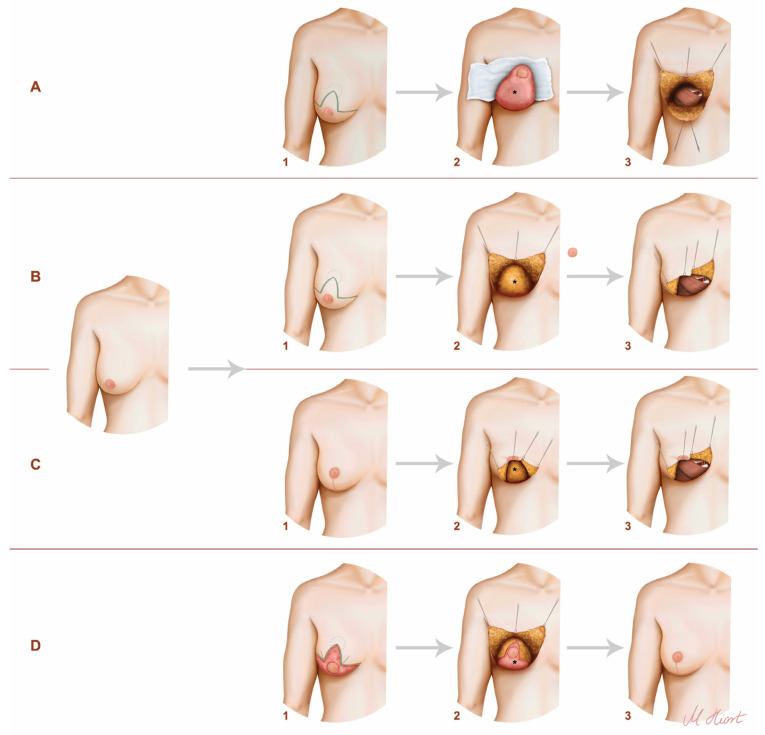
The techniques used in our clinic. The illustration on the left demonstrates the preoperative condition of the patients. (**A**) One-staged procedure with inferior pedicled NAC—1: preoperative markings; 2: intraoperatively; 3: exposure of the internal mammary vessels (IMV); *: inferior dermal pedical. (**B**) One staged procedure with free NAC skin graft—1: preoperative markings; 2: intraoperatively; 3: exposure of the IMV; *: breast tissue. (**C**) Two-staged procedure—mastopexy before mastectomy and reconstruction—1: breast after mastopexy/reduction; 2: mastectomy; 3: exposure of the IMV; *: breast tissue. (**D**) Two-staged procedure—mastopexy/reduction after mastectomy and reconstruction—1: de-epithelialized breast after reconstruction; 2: mastopexy/reduction; 3: postoperatively; *: DIEP flap.

All patients with grade 2 or 3 ptosis were treated with one of the following procedures (Figure 1).

### 2.2. Surgical Technique

All included breasts underwent both mastectomy and autologous breast reconstruction. The mastectomies were performed by multiple plastic surgeons at our institution. Wise pattern excision incision lines were marked preoperatively on a standing patient. The surgeries were performed in a two-team approach.

#### 2.2.1. One-Stage Procedures

- Pedicled nipple areola complex (NAC)

**Figure 2:**
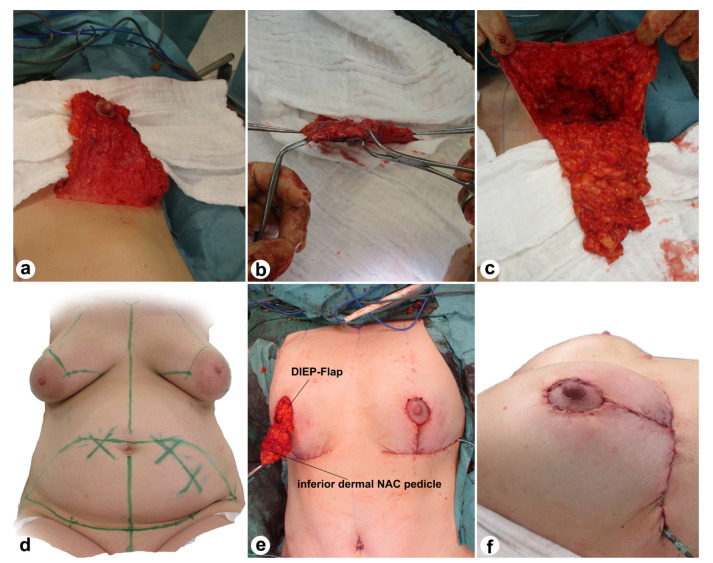
Intraoperative view (**a**–**c**,**e**) and markings (**d**) of the Wise pattern excision and the inferior dermal NAC pedicle. The last picture (**f**) demonstrates the intraoperative result after bilateral Wise pattern excision with pedicled NAC, mastectomy, and breast reconstruction with DIEP.

During this procedure, the mastectomy, autologous breast reconstruction, and mastopexy were executed as a one-stage procedure. A triangular inferior dermal pedicle was dissected with a retroareolar thickness of 0.5 cm and a thickness of 1 cm for the rest of the pedicle (Figure 2).

- Free skin graft

The Wise pattern excision lines are the same as above. However, during this procedure no inferior dermal flap was dissected. Instead, the NAC was grafted as a free skin graft.

#### 2.2.2. Two-Stage Procedures

- Breast reduction first

In this approach, patients received a breast reduction/mastopexy during the first surgery. The second surgery, which involved a mastectomy and autologous breast reconstruction, was performed 7.4 ± 3.8 months (range 4.6–16.7 months) after the reduction/mastopexy.

- Mastectomy and autologous breast reconstruction first

In this approach, the patient received a bilateral mastectomy and autologous breast reconstruction with a DIEP flap through inframammary fold incisions during the first surgery. The mastopexy was then performed 11 months after the first surgery. 

## 3. Results

Patient characteristics, surgery details, and postoperative complications are presented in Table 1 and Table 2.

### 3.1. One-Stage Procedures

#### 3.1.1. Pedicled Nipple Areola Complex (NAC) (Figure 3)

Five patients received 10 flaps with a mean surgery time of 320.8 ± 30.0 min. The time to ambulation for this group was 8.0 ± 2.0 days. Partial NAC necrosis occurred in two patients (three breasts, 30.0%), with one patient requiring a revision operation for necrosis excision (Figure 4). The third partial necrosis was managed conservatively. Both patients showed satisfactory results during follow-up. One breast (10.0%) required a revision due to arterial thrombosis, with the flap being saved after anastomosis revision. The NAC involved showed a partial necrosis (right breast Figure 4). Wound dehiscence occurred in two breasts (20.0%) of the same patient, and one patient (20.0%) experienced a donor site infection. No cases of fat necrosis were observed. 

**Figure 3:**
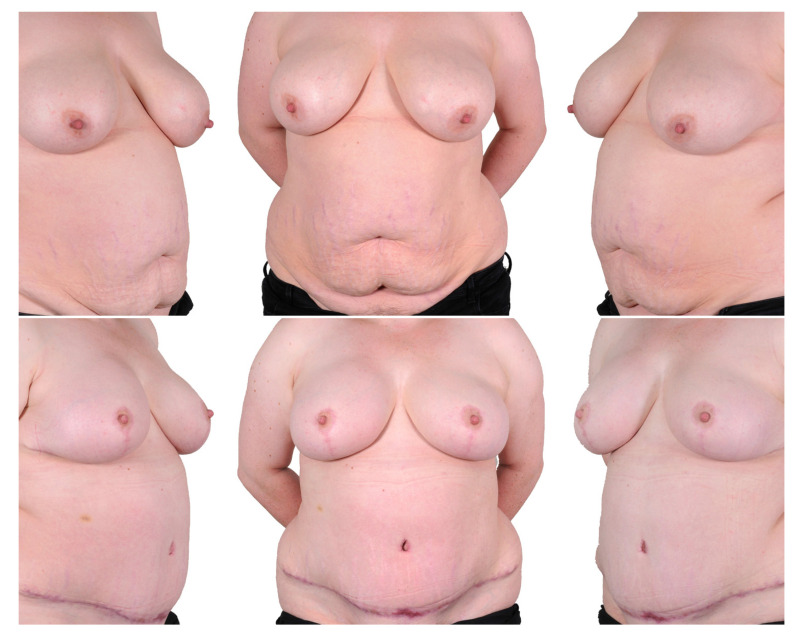
One-stage procedure—bilateral mastectomy, mastopexy with inferior dermal NAC pedical, DIEP. Top row: preoperatively; bottom row: postoperatively.

**Figure 4:**
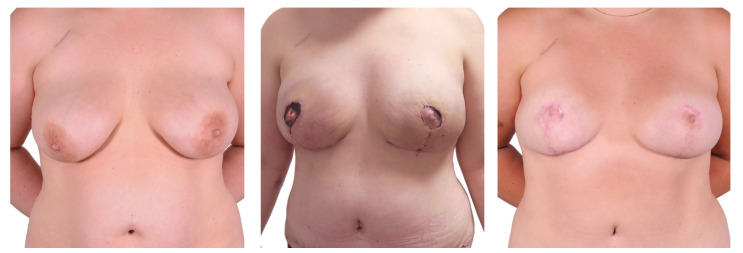
One-stage mastectomy, Wise pattern skin excision with inferior dermal NAC pedicle. From left to right: preoperatively/bilateral partial NAC necrosis 16 days after initial surgery/4 months after necrosis excision and 6 months after initial surgery.

#### 3.1.2. Free Skin Graft (Figure 5)

Three patients received a total of six flaps, with a mean surgery time of 309.0 ± 39.0 min. The time to ambulation for this group was 6.7 ± 1.2 days. One patient (two breasts, 33.3%) experienced a partial NAC necrosis which was managed conservatively. No anastomosis revision was required. One breast (16.7%) showed signs of fat necrosis. No wound dehiscence at the breast or donor site complications was observed.

**Figure 5:**
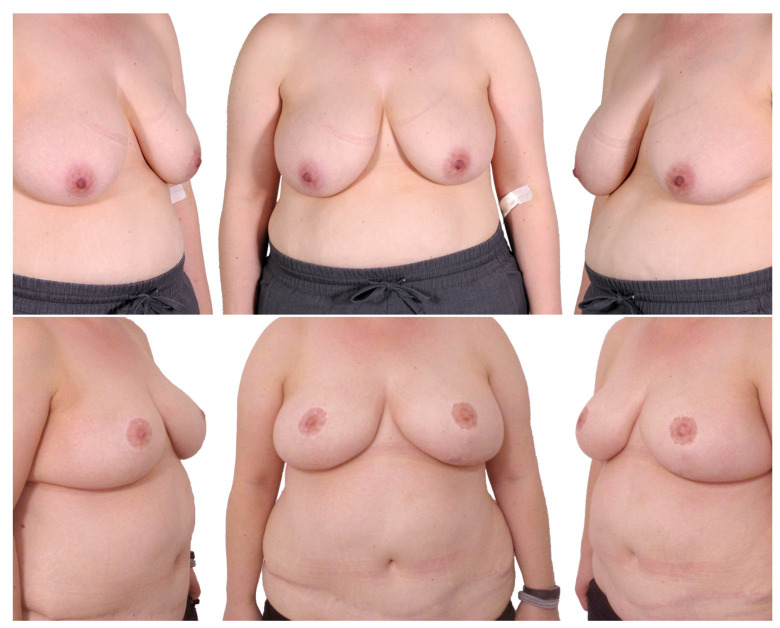
One-stage procedure—bilateral mastectomy, mastopexy with free skin graft, DIEP. Top row: preoperatively; bottom row: postoperatively.

### 3.2. Two-Stage Procedures

#### 3.2.1. Breast Reduction First (Figure 6)

Six patients received 11 flaps. In one case, the second flap was not transferred due to insufficient perfusion.

The total surgery time for the mastopexy was 125.0 ± 19.9 min, and for the bilateral mastectomy and autologous breast reconstruction it was 320.2 ± 69.9 min. The time to ambulation was 2.7 ± 1.2 days after the mastopexy and 6.4 ± 1.7 after the mastectomy and breast reconstruction. There were no cases of total or partial NAC necrosis. No anastomosis revision was necessary. Two breasts (18.0%) on two patients showed a wound dehiscence, and four patients (66.7%) experienced a donor site complication. Three of them showed a wound dehiscence and one patient had a donor site seroma and infection. 

**Figure 6:**
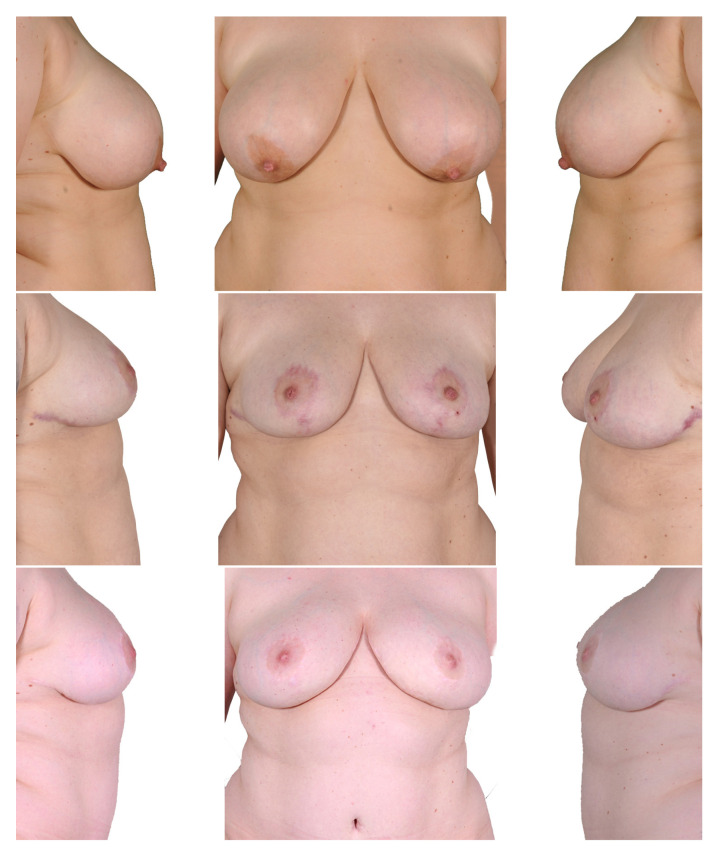
Two-stage procedure—1. breast reduction, 2. bilateral mastectomy and DIEP. Top row: preoperatively; middle row: postoperatively after breast reduction; bottom row: postoperatively after mastectomy and autologous breast reconstruction.

#### 3.2.2. Mastectomy and Autologous Breast Reconstruction First (Figure 7)

Only one patient underwent this procedure due to an unsatisfactory result after the initial surgery. There was no flap loss and no occurrence of total loss of the nipple areola complex. One breast (50%) showed signs of fat necrosis. No anastomosis revision was necessary. No breast wound dehiscence or donor site complication occurred in this case. 

**Figure 7:**
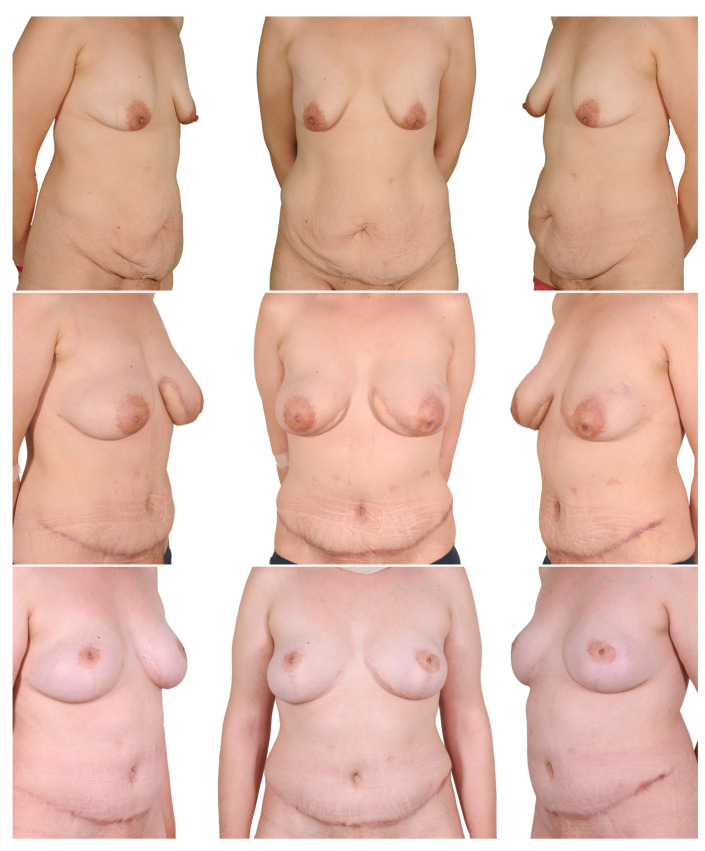
Two-stage procedure—1. bilateral mastectomy and DIEP, 2. mastopexy. Top row: preoperatively; middle row: postoperatively after mastectomy and autologous breast reconstruction; bottom row: postoperatively after mastopexy.

## 4. Discussion

Whether concerning post bariatric patients or patients with large and ptotic breasts, reconstructive surgeons face several challenges when planning a bilateral prophylactic mastectomy and autologous breast reconstruction. This group of patients has long been considered high-risk, due to an increased risk of NAC and skin flap necrosis [13,14]. A study of 2023 NSMs showed that patients with ptosis may exhibit poorer postoperative results [15].

The operative planning and decision-making process regarding one- or two-stage reconstruction and whether to use a pedicled or free NAC graft are crucial in achieving an aesthetically pleasing outcome. Although the primary goal is to remove at-risk breast tissue, the long-term cosmetic outcome has also been demonstrated to have a strong impact on patients’ quality of life [6,16,17,18,19,20,21]. The aim of this paper is to provide a review of the current literature on prophylactic mastectomy and autologous reconstruction in patients with large and ptotic breasts and an overview of the techniques used at our institution, which may help inform clinical decision-making and improve the quality of care for this challenging group of patients. According to the Consensus Conference of the Oncoplastic Breast Consortium on nipple-sparing mastectomy in women with cup size ≥ C and ptosis ≥ grade 2 without other risk factors for nipple necrosis, NSM can be performed with skin reduction and nipple–areola pedicles. This is regardless of the breast reconstruction technique, or with skin reduction and free-nipple grafting [22].

The method most frequently addressed in the literature for reconstruction of large and ptotic breasts is heterologous reconstruction using breast implants, either as a one-stage procedure using a permanent implant or as a two-stage procedure using a tissue expander which is later replaced by a permanent implant [8,23,24,25,26,27,28].

Despite rising evidence of higher long-term satisfaction and increased health-related quality of life (HR-QoL) linked with autologous procedures, the number of patients undergoing this kind of reconstruction has decreased in the United States [29].

Due to greater psychosocial and sexual well-being, more natural characteristics of the breast, and better performance in terms of postoperative radiation, however, autologous reconstruction remains the gold standard for certain cases and should be preferred if appropriate surgical skills and anatomical conditions are given.

The two-stage procedure has been discussed in the past [9,30]. Alperovic et al., showed that in patients with previous reduction mammaplasty or mastopexy, nipple-sparing mastectomy can be considered safe if one year or more has passed since surgery. In shorter time periods, they recommend selectively using indocyanine green (ICG) to evaluate perfusion of the mastectomy flap and NAC intraoperatively. Reconstructive outcomes of nipple-sparing mastectomy in patients with mastopexy or breast reduction in history are comparable to those who underwent nipple-sparing mastectomy only [31].

Komorowska-Timek et al., have also demonstrated the efficacy of ICG perfusion mapping in predicting tissue necrosis of the mastectomy flaps [32].

This can be used in cases of one-stage reconstruction with an inferior pedicle if vascularization of the NAC is uncertain. We suggest the conversion from pedicled NAC to free-skin graft if there is uncertainty about adequate vascularization.

Tondu et al., have also described a two-stage procedure of mastectomy and heterologous breast reconstruction, with the first stage being an inferior pedicle breast reduction with Wise pattern skin reduction and expander implantation [24]. A two-stage approach with a mastopexy following a mastectomy and free flap reconstruction was also described previously [33,34]. In 1985, Hester reported on breast reduction with a central pedicle [35].

DellaCroce et al., have conducted a study on 70 patients with large and ptotic breasts, in which they performed NSM and immediate autologous reconstruction initially followed by a second-staged mastopexy using a central pedicle [34]. They achieved high patient satisfaction with low complication rates and no cases of nipple–areola complex necrosis. The authors advocate for a two-stage procedure, especially because NAC repositioning requires a robust nipple–areola complex revascularization from an underlying well-perfused flap before interrupting the surrounding cutaneous blood supply of the NAC. We had a similar experience in the one case we performed using this technique, which resulted in a satisfied patient. This method could potentially be used not only in patients with large and ptotic breasts but also in patients with asymmetry or an unsatisfactory result after a DIEP flap reconstruction, as an alternative or additional tool to lipofilling. 

Due to the prolonged duration and increased complexity of the combined operations, Schneider et al., also advocate a two-stage procedure and recommend performing a mastopexy or breast reduction simultaneously with secondary procedures often required following free flap reconstructions [33]. Interestingly, we did not find an increased surgery time in one-stage procedures compared to two-stage procedures in our study. 

Despite the good results, the main disadvantage of two-stage procedures is the need for two major surgeries. Minimizing the number of operations would be desirable.

Several surgical techniques have been developed to address this challenge. Gibson presented a subcutaneous mastectomy technique using an inferior pedicle back in 1979 [36]. Woods discussed the topic of NSM and simultaneous mastopexy with inferior and superior pedicle in 1987 [37]. Kontos et al., presented a mastopexy technique with two skin flaps and a resulting horizontal scar in the middle of the breast as a one-stage procedure in combination with a mastectomy and immediate implant reconstruction [38]. The inferior dermal pedicle was also described by other authors as a basis for an implant-based reconstruction after mastectomy [27,39,40].

Surgical delay was also described to facilitate pedicled NSM and reconstruction in the ptotic breast [41].

The thickness of the mastectomy skin flap plays a crucial role for skin viability. Larson et al., stated that using the subcutaneous layer present in most breasts as a guide for elevating the skin flaps can achieve both oncologic safety and viable skin flaps [42].

Davies et al., reported higher skin-sparing mastectomy flap morbidity in breasts where the mastectomy was performed using Wise pattern or tennis pattern incisions. The main complication associated with the Wise incision was found to be wound dehiscence, especially at the T-junction (32%) [43].

Egan et al., highlighted that hypopigmentation, depigmentation, partial graft loss and loss of nipple projection are complications which are not rare after free-nipple areolar grafting. However, these complications were not statistically correlated with NAC aesthetic or overall aesthetic rate in their study [44].

There are only a few reports on one-stage autologous reconstruction in large and ptotic breasts. In a study by Pontell et al., bilateral NSM with a simultaneous reduction mastopexy and immediate reconstruction using a DIEP flap or expander were performed in eight patients with large ptotic breasts. To decrease the risk of NAC necrosis, wide, inferior, epithelialized pedicles were used for reconstruction [8]. However, they reported on a high number of nicotine-associated NAC necrosis occurrences while observing a small sample size. In a recent study of Rose et al., buried autologous breast reconstruction (BARB) flaps were compared with non-buried flaps in one-stage procedures after mastectomy. In the case of large and ptotic breasts, Wise pattern reduction mastectomy was performed with or without nipple preservation on an inferior pedicle or as a free-nipple graft. The authors concluded that buried free flaps are safe and reliable with lower revision rates than those with cutaneous paddles. They recommended single-stage reconstruction regardless of breast characteristics [10]. 

Advances in mastectomy techniques lead to better aesthetic results. Reconstructive options have improved substantially, making it possible to achieve a good cosmetic result in women with large and ptotic breasts. The choice of the most suitable technique for prophylactic mastectomy and reconstruction in this patient group depends not only on the surgeon’s experience and preference but also on various factors, such as the patient’s anatomy, comorbidities and aesthetic goals. The oncological safety of a pedicled NSM compared to a free full-thickness skin graft can be debated, since the former preserves a small amount of breast tissue, although located in an easily palpable area. 

Avoiding complications such as necrosis of the nipple–areola complex or skin flap necrosis remains one of the main concerns when performing NSM on ptotic breasts. Careful surgical planning is crucial to achieving optimal outcomes.

One limitation of the study was its retrospective nature, as well as the small number of cases included.

## 5. Conclusions

There are a number of different options for performing bilateral prophylactic mastectomy and autologous breast reconstruction in patients with large and ptotic breasts. Mastopexy can be performed before, with, or after mastectomy and autologous reconstruction with good surgical outcomes. Larger studies with longer follow-up periods are needed to fully assess the oncological safety and patient satisfaction associated these procedures.

## Figures and Tables

**Table 1:** Patient characteristics (Mean ± SD).

	One-Stage		Two-Stage	
	NAC		BR/Mastopexy	
	Pedicled	Free	First	Last
No. of flaps				
Total	10	6	11	2
Unilateral	0	0	1	0
Bilateral	10	6	10	2
No. of patients				
Total	5	3	6	1
Unilateral	0	0	1	0
Bilateral	5	2	5	1
Follow-up (months)				
	5.2 ± 1.5	3.6 ± 1.8	10.8 ± 5.1	26.9
Age (years)				
	40.7 ± 8.9	39.8 ± 4.9	39.9 ± 13.8	37.6
BMI (kg/m^2^)				
	29.4 ± 4.3	28.0 ± 2.0	31.7 ± 3.1	20.0
Comorbidities				
Nicotine	0 (0.0)	0 (0.0)	1 (16.7)	0 (0.0)
Diabetes mellitus	0 (0.0)	0 (0.0)	0 (0.0)	0 (0.0)
Arterial hypertension	0 (0.0)	0 (0.0)	2 (33.3)	0 (0.0)
Hyperthyroidism	3 (60.0)	1 (33.3)	1 (16.7)	0 (0.0)
Anticoagulation	1 (20.0)	0 (0.0)	0 (0.0)	0 (0.0)
Radiation	1 (10.0)	0 (0.0)	3 (27.3)	0 (0.0)
BRCA	5 (100.0)	3 (100.0)	6 (100.0)	1 (100.0)
Breast cancer	2 (40.0)	0 (0.0)	2 (33.3)	0 (0.0)

SD: standard deviation; NAC: Nipple Areola Complex; BR: Breast reduction; first: mastopexy performed before mastectomy and autologous reconstruction; last: mastopexy performed after mastectomy and autologous reconstruction.

**Table 2:** Surgery and postoperative details (Mean ± SD).

	One-Stage		Two-Stage	
	NAC		BR/Mastopexy	
	Pedicled	Free	First	Last
Total surgery time (min)				
Mastectomy + DIEP + BR	320.8 ± 30.0	309.0 ± 39.0	-	-
BR	-	-	125.0 ± 19.9	116.0
Mastectomy + DIEP	-	-	320.2 ± 69.9	552.0
Flap ischemia time (min)				
	51.7 ± 25.0	44.0 ± 14.1	45.8 ± 20.1	43.0
Length of stay (d)				
Mastectomy + DIEP + BR	8.0 ± 2.0	6.7 ± 1.2	-	-
BR	-	-	2.7 ± 1.2	2.0
Mastectomy + DIEP	-	-	6.4 ± 1.7	9.0
Months between surgeries				
	-	-	7.4 ± 3.8	11.0
Anastomosis revision (%)				
	1 (10.0)	0 (0.0)	0 (0.0)	0 (0.0)
Flap loss (%)				
total	0 (0.0)	0 (0.0)	0 (0.0)	0 (0.0)
partial	0 (0.0)	0 (0.0)	0 (0.0)	0 (0.0)
NAC loss (%)				
total	0 (0.0)	0 (0.0)	0 (0.0)	0 (0.0)
partial	3 (30.0)	2 (33.3)	0 (0.0)	0 (0.0)
Total flap loss (%)				
	0 (0.0)	0 (0.0)	0 (0.0)	0 (0.0)
Partial flap loss (%)				
	0 (0.0)	0 (0.0)	0 (0.0)	0 (0.0)
Fat necrosis (%)				
	0 (10.0)	1 (16.7)	0 (0.0)	1 (50.0)
Donor site complication (%)				
	1 (20.0)	0 (0.0)	4 (66.7)	0 (0.0)
Wound dehiscence breast (%)				
	2 (20.0)	0 (0.0)	2 (18.2)	0 (0.0)

SD: standard deviation; NAC: Nipple Areola Complex; BR: Breast reduction; first: mastopexy performed before mastectomy and autologous reconstruction; last: mastopexy performed after mastectomy and autologous reconstruction.

## Data Availability

Data available on request.
